# Progesterone (P4) ameliorates cigarette smoke-induced chronic obstructive pulmonary disease (COPD)

**DOI:** 10.1186/s10020-024-00883-y

**Published:** 2024-08-13

**Authors:** Bin Xie, Qiong Chen, Ziyu Dai, Chen Jiang, Xi Chen

**Affiliations:** 1 Center of Respiratory Medicine, Xiangya Hospital, Central South University, Changsha, Hunan 410008 China; 2 Departement of Geriatrics, Respiratory Medicine, Xiangya Hospital, Central South University, Changsha, 410008 China; 3 National Key Clinical Specialty, Branch of National Clinical Research Center for Respiratory Disease, Xiangya Hospital, Central South University, Changsha, Hunan 410008 China; 4 National Clinical Research Center for Geriatric Disorders, Xiangya Hospital, Central South University, Changsha, 410008 China

**Keywords:** Chronic obstructive pulmonary disease (COPD), Oxidative injury, Mitochondrial dysfunction, Airway epithelium, Progesterone (P4)

## Abstract

**Background:**

Chronic obstructive pulmonary disease (COPD) is a chronic inflammatory lung disease associated with high morbidity and mortality worldwide. Oxidative injury and mitochondrial dysfunction in the airway epithelium are major events in COPD progression.

**Methods and results:**

The therapeutic effects of Progesterone (P4) were investigated in vivo and in vitro in this study. In vivo, in a cigarette smoke (CS) exposure-induced COPD mouse model, P4 treatment significantly ameliorated CS exposure-induced physiological and pathological characteristics, including inflammatory cell infiltration and oxidative injury, in a dose-dependent manner. The c-MYC/SIRT1/PGC-1α pathway is involved in the protective function of P4 against CS-induced COPD. In vitro, P4 co-treatment significantly ameliorated H_2_O_2_-induced oxidative injury and mitochondrial dysfunctions by promoting cell proliferation, increasing mitochondrial membrane potential, decreasing ROS levels and apoptosis, and increasing ATP content. Moreover, P4 co-treatment partially attenuated H_2_O_2_-caused inhibition in Nrf1, Tfam, Mfn1, PGR-B, c-MYC, SIRT1, and PGC-1α levels. In BEAS-2B and ASM cells, the c-MYC/SIRT1 axis regulated P4’s protective effects against H_2_O_2_-induced oxidative injury and mitochondrial dysfunctions.

**Conclusion:**

P4 activates the c-MYC/SIRT1 axis, ameliorating CS-induced COPD and protecting both airway epithelial cells and smooth muscle cells against H_2_O_2_-induced oxidative damage. PGC-1α and downstream mitochondrial signaling pathways might be involved.

**Supplementary Information:**

The online version contains supplementary material available at 10.1186/s10020-024-00883-y.

## Introduction

Chronic obstructive pulmonary disease (COPD) is a chronic inflammatory lung disease associated with high morbidity and mortality worldwide. COPD is characterized by a progressive deterioration of lung function and an abnormal inflammatory reaction of the lungs to factors such as cigarette smoking, biofuels, and air pollution (Belvisi et al. 2004; Barnes 2013). However, due to the complexity of the COPD etiology, existing clinical treatments cannot successfully impede COPD progression.

The pathogenesis of COPD encompasses various factors, including oxidative stress, inflammation, protease-antiprotease imbalance, apoptosis, and immunity (Tse and Tseng 2014; Stefano et al. 2004; Barnes 2014). Among them, oxidative stress exerts an essential effect on COPD pathogenesis, mediating various signal transduction pathways, participating in gene expression regulation, and affecting lung inflammation and airway remodeling (Tse and Tseng 2014; Stefano et al. 2004; Barnes 2014). Stimulation such as inhaled cigarette smoke (CS), primarily encounters the epithelial lining of the lungs, where it induces oxidative stress through acute effects of its reactive components and intracellular ROS (reactive oxygen species) production by mitochondria (Bohr et al. 2002; Bohr 2002; Barnes 2006; Wu et al. 2013; Afonso et al. 2007; Toorn et al. 2009). The various ways in which mitochondria protect themselves and the cell from oxidative injury include the production of antioxidant scavengers, the regulation of oxidative phosphorylation for ATP formation, and the exchange of mitochondrial DNA via fusion-fission events (Afonso et al. 2007; Berman et al. 2008; Chan 2006; Meissner 2007). Excessive oxidative stress and/or imbalance or exhaustion of key mitochondrial fission and fusion markers, including Dynamin-related protein 1 (Drp1), mitochondrial fission 1 protein (Fis1), mitofusin (Mfn1 and Mfn2), optic nerve atrophy 1 (OPA1) and mitochondrial transcription factor A (Tfam), can cause mitochondrial damage and cristae formation disorders and abnormalities (Jendrach et al. 2005). Therefore, oxidative injury and mitochondrial dysfunction in airway epithelium could be pivotal events in COPD progression.

Notably, low levels of progestins in post-menopausal women can lead to insufficient natural breathing stimulation, making women prone to respiratory dysfunction. Previously, medroxyprogesterone, a progesterone (P4) derivative, has been reported to improve the respiratory function of COPD patients (Saaresranta et al. 2005; Wagenaar et al. 2002, 2003). Nerve cell studies have found that P4 can alleviate mitochondrial dysfunction caused by oxidative stress (Robertson et al. 2006; Gaignard et al. 2016; Cai et al. 2015), restore mitochondrial membrane potential (Qin et al. 2015), increase the activity of respiratory chain complex I, and enhance the function of oxide disproportionation (Gonzalez Deniselle et al. 2012). In breast cancer cells and uterine leiomyoma tissues, P4 acts on the progesterone receptor (PGR) to increase mitochondrial membrane potential and ATP production, contributing to the restoration of mitochondrial functions (Feng et al. 2014; Dai et al. 2013). Given the critical roles of oxidative injury and mitochondrial dysfunction in COPD pathogenesis, it is reasonably deduced that P4 might protect airway epithelium from oxidative injury and mitochondrial dysfunction, thereby alleviating the progression of COPD.

After binding to P4, the activated PGR translocates to the nucleus, where it can bind to specific progesterone response elements in the promoter region of the *c-MYC* gene, thereby promoting its transcription (Dinh et al. 2019; Moore et al. 1997a, b). Reportedly, c-MYC expression in the lung tissue of emphysema mouse models is significantly reduced (Muyal et al. 2014), and it is believed that c-MYC participates in the occurrence and progression of COPD. C-MYC can combine with the promoter of *Sirtuin 1* (*SIRT1*) to promote *SIRT1* expression (Yuan et al. 2009). SIRT1 is an important deacetylase that can mediate the deacetylation of the main regulator of mitochondrial biosynthesis, peroxisome proliferators activated receptor gamma co-activator 1 alpha (PGC-1α) to activate PGC-1α. Activated PGC-1α subsequently regulates the expression of downstream mitochondrial function-related genes such as *Nrf1*, *Nrf2*, *Tfam*, *Mfn1*, and *Drp1* (Dabrowska et al. 2015), and plays an important role in mitochondrial oxidative stress, oxidative phosphorylation, and cellular energy metabolism. Studies have shown that the expression of SIRT1 is significantly reduced in the lung tissue of COPD patients (Baker et al. 2016), while PGC-1α is compensatively increased in patients with mild COPD but severely decreased in patients with severe COPD (Li et al. 2010).

Considering these previous findings, it is hypothesized that P4 might protect airway epithelium against oxidative injury and mitochondrial dysfunction through the c-MYC/SIRT1/PGC-1α signaling. First, cigarette smoke (CS)-induced COPD model was established in mice and the therapeutic effects of different concentrations of P4 were investigated in vivo. Moreover, the pathogenesis of COPD is heavily influenced by oxidative stress. Inhaling cigarette smoke (CS) induces oxidative stress, resulting in the excessive production of ROS (Schunemann et al. 1997), which subsequently causes airway epithelial injury and the accumulation of inflammatory mediators, exacerbating COPD (Eeden and Sin 2013; Donnelly and Barnes 2006). In COPD, changes such as epithelial metaplasia and a rise in the amount of airway smooth muscle are linked to reduced airflow and diminished pulmonary function (Abohalaka 2023). Furthermore, it has been demonstrated that oxidative stress impacts the balance and restoration of both airway epithelial cells and smooth muscle cells within COPD (Esteves et al. 2021). Therefore, H_2_O_2_ stimulation was conducted on lung bronchus epithelial cell BEAS-2B and airway smooth muscle cells (ASM cells), and the in vitro effects of P4 treatment were investigated. The changes in Nrf1, Tfam, Mfn1, PGR-B, c-MYC, SIRT1, and PGC-1α were also examined. Finally, the dynamic effects of the c-MYC/SIRT1 axis on H_2_O_2_-stimulated BEAS-2B and ASM cells were evaluated.

## Materials and methods

### Establishment of COPD mouse model

A total of 30 male C57BL/6J mice (8 weeks old, about 20 g) were purchased from SLAC Laboratory Animal Co. LTD. (Changsha, China). All experimental procedures were approved by the Ethics Committee of Xiangya Hospital of Central South University. The COPD model was established by cigarette smoke (CS) exposure. Mice were randomly divided into 5 groups: control, CS, CS + P4- low dose CS + P4-moderate dose, and CS + P4-high dose groups. In CS and CS + P4 groups, mice were exposed to tobacco smoke the equivalent of five cigarettes, 4 times daily for 30 min, 6 days per week for 15 weeks (Wang et al. 2021). From week 8, CS + P4 group mice received inhalation of P4 (0.03, 0.1, and 0.3 mg/L) for 30 min (Fei et al. 2017) per day using an ultrasonic nebulizer (spray speed 0.4 mL/min YUYUE, Zhenjiang, China). The dose of P4 inhalation administration was about 18, 60 and 180 µg/kg in low, moderate, and high dose groups, respectively. The control and CS groups were inhaled with saline for 30 min per day. The mice were sacrificed on the day after the last CS exposure. The lung tissues and bronchoalveolar lavage fluid (BALF) were harvested as previously described (Peng et al. 2018; Cui et al. 2022).

### Hematoxylin and eosin (H&E) staining

After regular fixing and embedding procedures, the lung tissues were sectioned at a thickness of 4 μm and deparaffinized with xylene. Finally, the sections underwent HE staining (Zhou and Moore 2017) and were observed under an optical microscope.

### Measurement of oxidative parameters

About 50 mg mouse lung tissues were lyzed in cold saline (1:9 v/v) using a tissue homogenizer at 60 Hz for 2 min (Beyotime, Shanghai, China). After centrifuging for 10 min at 2500 rpm, the supernatant was first collected for protein concentration determination using a BCA protein assay kit (Beyotime). The level of malondialdehyde (MDA) and activity of catalase (CAT) and superoxide dismutase (SOD) in the lung tissues homogenate was subsequently determined using an MDA assay kit (TBA method, A003-1-2, Nanjing Jiancheng Bioengineering Institute, Nanjing, China), CAT assay kit (visible light, A007-1-1, Nanjing Jiancheng), and SOD assay kit (WST-1 method, A001-3-2, Nanjing Jiancheng) respectively.

### Cell culture and cell treatment

The human lung bronchus epithelial cell line BEAS-2B (CRL-9609) was procured from ATCC (Manassas, VA, USA) and cultured using a Bronchial Epithelial Cell Growth Medium Bullet Kit (Catalog No. CC-3170; Basel, Switzerland). Human airway smooth muscle cells (ASM cells, PCS-130-011) were obtained from ATCC and cultured using the Vascular Cell Basal Medium (PCS-100-030, ATCC) supplemented with a Vascular Smooth Muscle Cell Growth Kit (PCS-100-042, ATCC). All cells were cultured at 37 °C in 5% CO_2_. For inducing oxidative injury, cells were exposed to 200 µmol/L H_2_O_2_ (Aladdin) for 16 h; for progesterone protection, cells were pre-treated with 0.5 µmol/L P4 (S1705, Selleck Chemicals, TX, USA), for 30 min and then co-treated with H_2_O_2_ for 16 h.

### Cell transfection

Cells were first plated into 6-well plates (Corning, NY, USA) and grown to a cell density of 60%. Transfection of si-c-MYC (50 nM), or pcDNA3.1/SIRT1 (2 µg) and corresponding negative controls (si-NC and vector; GenePharma, Shanghai, China) was performed according to the instructions of Lipofectamine 3000 (Invitrogen, CA, USA). After 48 h, the cells were collected for subsequent experiments. The sequence of siRNA and primer for vector construction is presented in Table S1.

### MTT assay detecting cell proliferation

Cells were seeded into a 96-well plate and placed in an incubator at 37 °C, 5% CO_2_ for 32 h and then treated with H_2_O_2_ or P4 for 16 h (48 h after cell seeding) .MTT (ST316, 0.5 mg/mL, Beyotime, Shanghai, China) was added to each well for 4 h in light-deprived conditions. After the treatment, the supernatant was discarded, and 150 µl of dimethyl sulfoxide (DMSO, Aladdin) was added to each well. The absorbance at 450 nm was measured with a microplate reader.

### Mitochondrial membrane potential (ΔΨm) determined by JC-1 probe

Cells, transfected or treated, were cultured in six-well plates, incubated with 1 mL of pre-warmed JC-1 staining solution (5 µg/mL) (M8650, Solarbio, Beijing, China) at 37 °C for 20 min, and washed thrice with PBS. The cells were collected, and the fluorescence intensity was measured by flow cytometry (Novocyte, CA, USA). Mitochondrial membrane depolarization is manifested as a decrease in the rate of the Q2 quadrant (indicating the red inflorescence).

### Mitochondrial ROS determination

Mitochondrial ROS levels were estimated using MitoSOX™ Green Mitochondrial Superoxide Indicator (M36005; Thermo Fisher Scientific, MA, USA). The intensity of fluorescence was measured by flow cytometry (Novocyte).

### ATP content

An ATP detection kit (S0026, Beyotime) was used to detect the ATP content as directed by the manufacturer. After treatment, BEAS-2B cells or ASM cells were collected and lysed. After centrifuging at 12,000 g for 5 min at 4 °C, 20 µL of the supernatant was mixed with 100 µL ATP working solution and the absorbance was measured with a microplate reader (PerkinElmer, FL, USA). The ATP content was quantified with reference to the standard curve.

### Transmission electron microscope (TEM) observation

TEM analysis of mitochondria was performed as previously described (Li et al. 2023). After different treatments, ASM cells were collected and fixed by 2.5% glutaraldehyde and further post-fixed in osmium tetroxide. After dehydration and embedding, samples were cut into 0.1 μm-thick cross-sections by an ultramicrotome (Reichert-Jung, German) and picked up on copper electron microscope grids and counterstained with uranyl acetate and lead citrate before being observed by TEM (Hitachi, Tokyo, Japan).

### Immunoblotting

Cell and tissue protein samples were extracted as previously described (Wang et al. 2021). The proteins were subsequently separated on an SDS polyacrylamide gel and transferred to a polyvinylidene fluoride membrane. The cell membrane was blocked with 5% skim milk to prevent non-specific bindings for 1 h and then incubated with the primary antibody at 4 °C overnight. The following primary antibodies were used: Nrf1 (12482-1-AP; Proteintech, Wuhan, China), Tfam (22586-1-AP, Proteintech), Mfn1 (66776-1-Ig, Proteintech), PGR-B (25871-1-AP, Proteintech), c-MYC (10828-1-AP, Proteintech), SIRT1 (60303-1-Ig, Proteintech), PGC-1α (66369-1-AP, Proteintech). After overnight incubation with primary antibodies at 4℃, the membranes were further incubated with secondary antibodies (SA00001-1, SA00001-2, Proteintech). The blot signaling was visualized by the enhanced chemiluminescent substrate kit (P0018FS, Beyotime) and observed by the automatic chemiluminescent imaging system (Tannon, Shanghai, China).

### Luciferase reporter assay

To assess the impact of PGR-B on the *c-MYC* transcription, a PGR-B-overexpressing plasmid (PGR OE), plasmid contained a short hairpin RNA targeting *PGR-B* (sh-PGR), and luciferase reporter plasmid psiChesk2 contained wild type or mutant type *c-MYC* promoter fragment (*c-MYC* pro -wt or *c-MYC* pro -mut) were constructed. Then, the reporter plasmid was co-transfected with PGR-OE or sh-PGR into BEAS-2B and ASM cells. After 24 h, the cells were harvested for luciferase reporter assay. Firefly and Renilla luciferase activity was quantified using a Dual-Luciferase Reporter Assay System (Promega, Madison, WI, USA). The ratio of firefly luciferase to Renilla activity was determined for each sample.

### PGC-1α acetylation status analysis

BEAS-2B and ASM cells were transfected with si-c-MYC, si-NC (negative control), SIRT1-overexpressing vector, or empty vector for 48 h. Then, cells were collected for co-immunoprecipitation (co-IP). Cells were lysed in IP lysis buffer (Thermo Fisher Scientific, MA, USA) containing protease inhibitors (Roche, Basel, Switzerland). After centrifuging at 10,000 x g for 5 min at 4℃, One part of the supernatant was saved as input. The rest supernatant was used for co-IP. The protein A/G agarose beads (Beyotime) were incubated with anti-PGC-1α antibody (sc-518025, Santa Cruz Biotechnology, Santa Cruz, CA, USA, ) or anti-IgG (Negative control, 65208-1-Ig, Proteintech) for 1 h at room temperature with rotation. After washed with TBS, the beads were further incubated with the cell lysate supernatant overnight at 4 °C with rotation. The beads were washed and eluted with elution buffer (Beyotime). The eluted proteins were subjected to immunoblotting analysis as above mentioned to determine PGC-1α and acetylated PGC-α levels using an anti-acetyl-lysine antibody (PTM-101, PTM Bio, Hangzhou, China).

### Statistical analysis

A student’s t-test was used for data with only two groups. An analysis of variance (ANOVA) test was used for data containing more than two groups, followed by the Tukey post-hoc test. All statistical analyses were performed using SPSS 22.0 (IBM, Armonk, NY, USA). The diagrams were drawn using GraphPad 8.0 (San Diego, CA, USA).

## Results

### P4-protected CS-induced COPD mouse model

**Fig. 1:**
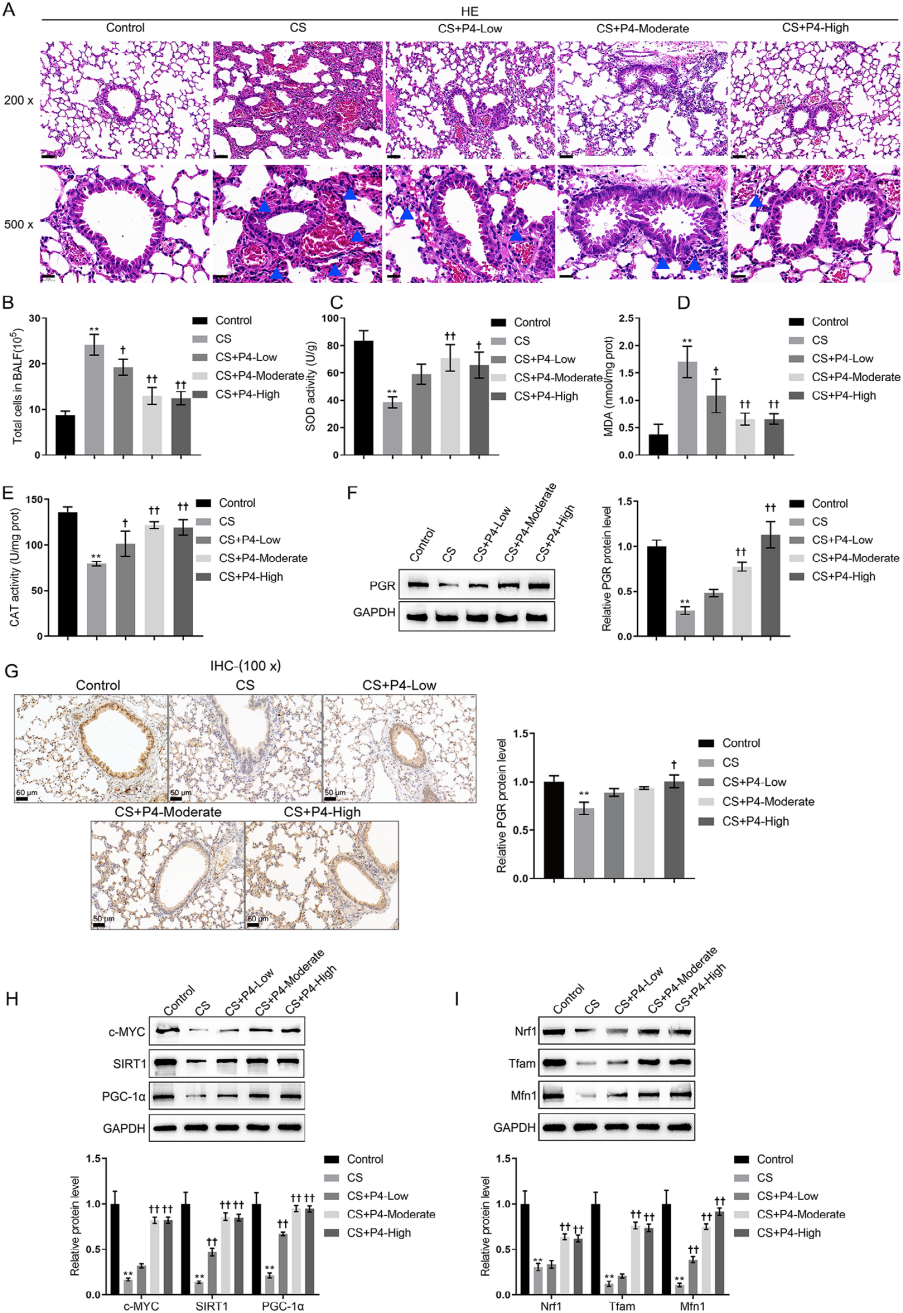
Progesterone (P4) protected CS-induced COPD mouse model. (**A**) The histological changes of lung tissues from control, CS, CS + P4-low, -moderate, and -high (inhalation of (0.03, 0.1, and 0.3 mg/L P4) group mice were observed by HE staining; the blue arrows indicated the infiltration of inflammatory cells. The scale bar is 50 μm for 200x and 20 μm for 500x. (**B**) The total cell number in BALF was counted. *n* = 6. (**C**-**E**) The level of MDA and the activity of SOD and CAT in mouse lung tissues homogenate were determined. (**F**-**G**) The protein levels of PGR-B in mouse lung tissues were determined using Immunoblotting and IHC staining. The scale bar is 50 μm. The relative expression of PGR-B was shown in the right panel of G. (**H**) and (**I**) The protein levels of c-MYC, SIRT1, PGC-1α, Nrf1, Tfam, and Mfn1 using Immunoblotting. *n* = 3. ** *p* < 0.01 compared to control group, † *p* < 0.05, †† *p* < 0.01 compared to CS group

Firstly, concerning the protective function of P4 in vivo, the CS-induced COPD mouse model was established, and low-, moderate-, and high-concentration P4 treatment was applied. A safety evaluation of P4 inhalation on male mice was performed. Healthy male mice were subjected to nebulized administration of low, moderate, and high concentrations of P4 for 8 weeks, and side effects during and after the treatment period were monitored and evaluated. The results showed that no significant changes in body weight, lung, liver, or spleen were observed across all groups after 8 weeks of P4 nebulization at varying doses, suggesting that the administration of P4, even at the highest dose tested, did not produce noticeable adverse effects in male mice (Fig.S1A-D). Next, in CS-exposed mice, inflammatory cells (blue arrows indicated) infiltrated into airway and lung tissues, and CS-induced lung injury was effectively reversed by P4 treatment (Fig. 1A); as shown in Fig. 1A, moderate- and high-concentration of P4 treatment restored the lung histopathology of the model mice to approximate that of the normal control group. Besides, increased numbers of total cells were observed in BALF from CS-exposed mice, whereas P4 treatment significantly reduced the numbers of total cells in BALF in a concentration-dependent manner (Fig. 1B). The anti-oxidative enzymes SOD and CAT’s activity was reduced in COPD lung tissues and upregulated by the P4 treatment. Correspondingly, the level of MDA was increased in COPD lung tissues and reduced by P4 treatment (Fig. 1.C-E). The c-MYC/SIRT1/PGC-1α pathway was also determined in the lung tissues. Most of the physiological functions of P4 are mediated through its receptor, PGR (Lee et al. 2006). Therefore, the expression of PGR-B was first determined. As depicted in Fig. 1F, CS exposure reduced PGR-B expression which could be reversed by P4 treatment. Moreover, PGR-B expression was further elucidated through IHC staining. These results demonstrated that both bronchus epithelial cells and smooth muscle cells in COPD mice lungs expressed the PGR-B which were downregulated by CS-exposure and reversed by P4 treatment (Fig. 1G). As shown by Fig. 1H and I, CS-exposure reduced c-MYC, SIRT1, PGC-1α, Nrf1, Tfam and Mfn1 expression, which could be reversed by P4 treatment. Consistently, P4 treatment reversed the levels of these factors in a dose-dependent manner.

### P4 increases epithelial cells and smooth muscle cells’ function and protects them from H_2_O_2_-induced oxidative injury

**Fig. 2:**
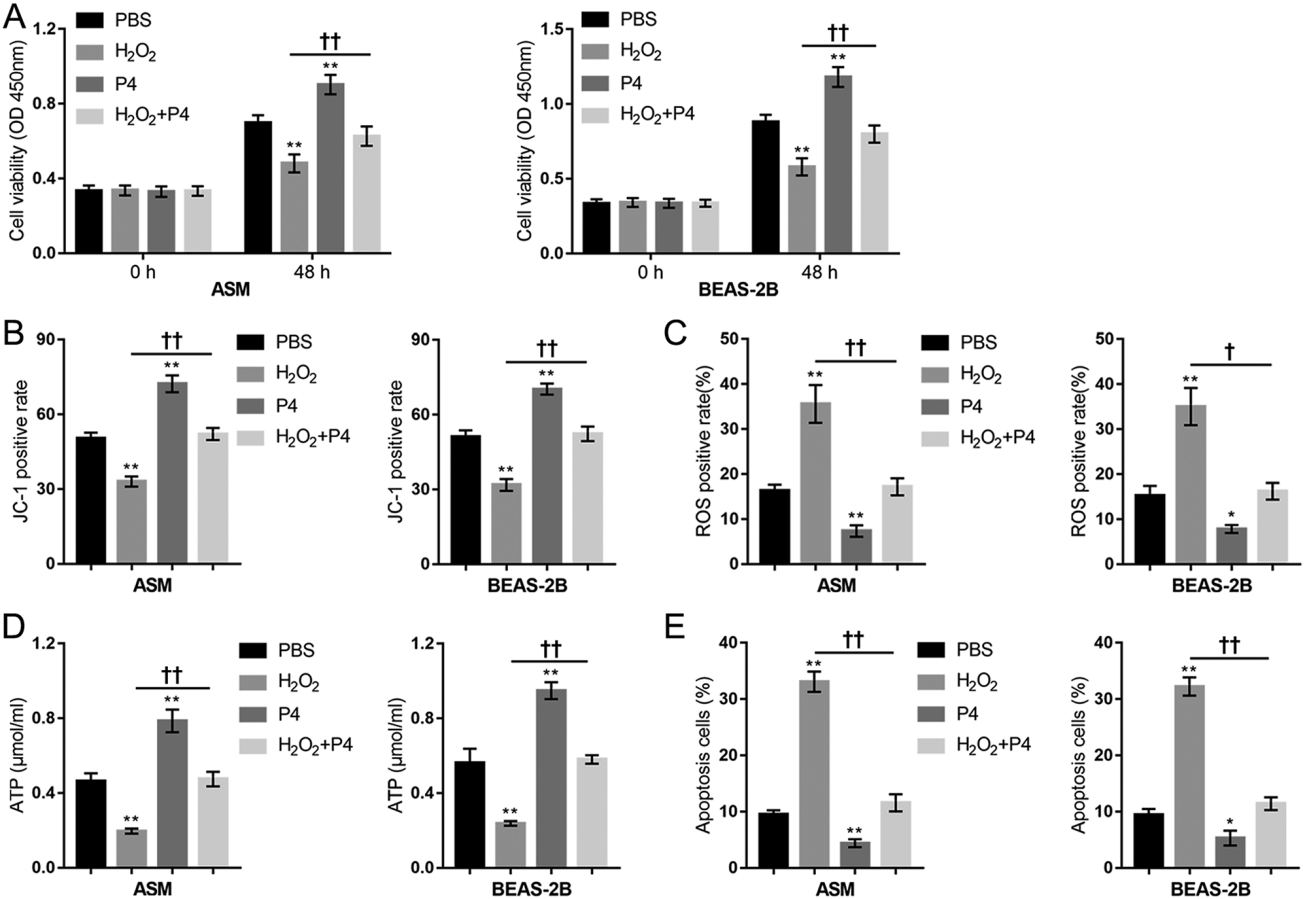
P4 protects lung epithelial cells and smooth muscle cells from H_2_O_2_ -induced oxidative injury. BEAS-2B and ASM cells were divided into four groups: PBS (control), H_2_O_2_, P4, and H_2_O_2_ + P4; cells were treated or co-treated as described and examined for cell viability using MTT assay (**A**); mitochondrial membrane potential by JC-1 staining (**B**); mitochondrial ROS by MitoSOX Green followed by Flow cytometry (**C**); ATP content using ATP Detection Kit (**D**); the apoptosis rate using Annexin-V/PI staining (**E**). *N* = 3, * *p* < 0.05, ** *p* < 0.01 compared to PBS group, † *p* < 0.05, †† *p* < 0.01 compared to H_2_O_2_ group

As mentioned above, CS causes oxidative stress, which produces excessive ROS (Schunemann et al. 1997), leading to airway epithelium injury and inflammatory mediator accumulation, thereby exacerbating COPD (Eeden and Sin 2013; Donnelly and Barnes 2006). The usage of human source cell lines was driven by the intent to explore the translational aspects of the findings from the mouse model to potential human therapeutic applications, making the research results relevant and applicable to human physiology. Therefore, the human lung bronchus epithelial cell line BEAS-2B and human airway smooth muscle cells were used in this study. H_2_O_2_-stimulated cells were used for in vitro investigations. Firstly, BEAS-2B and ASM cells were divided into four groups: PBS (control), H_2_O_2_, P4, and H_2_O_2_ + P4; P4 protection against H_2_O_2_-induced oxidative injury was investigated. H_2_O_2_ treatment induced inhibition in cell proliferation (Fig. 2A), a reduction in ΔΨm (Fig. 2B, Fig.S2B), increased mitochondrial ROS production (Fig. 2C, Fig.S2A), decreased cellular ATP content (Fig. 2D), and increased apoptosis (Fig. 2E, Fig.S2C), indicating H_2_O_2_-induced mitochondrial dysfunction and oxidative injury. Contrariwise, P4 treatment alone increased cell proliferation and mitochondrial function and reduced the baseline apoptosis rate; more importantly, P4 co-treatment attenuated H_2_O_2_-induced changes (Fig. 2A-E).

**Fig. 3:**
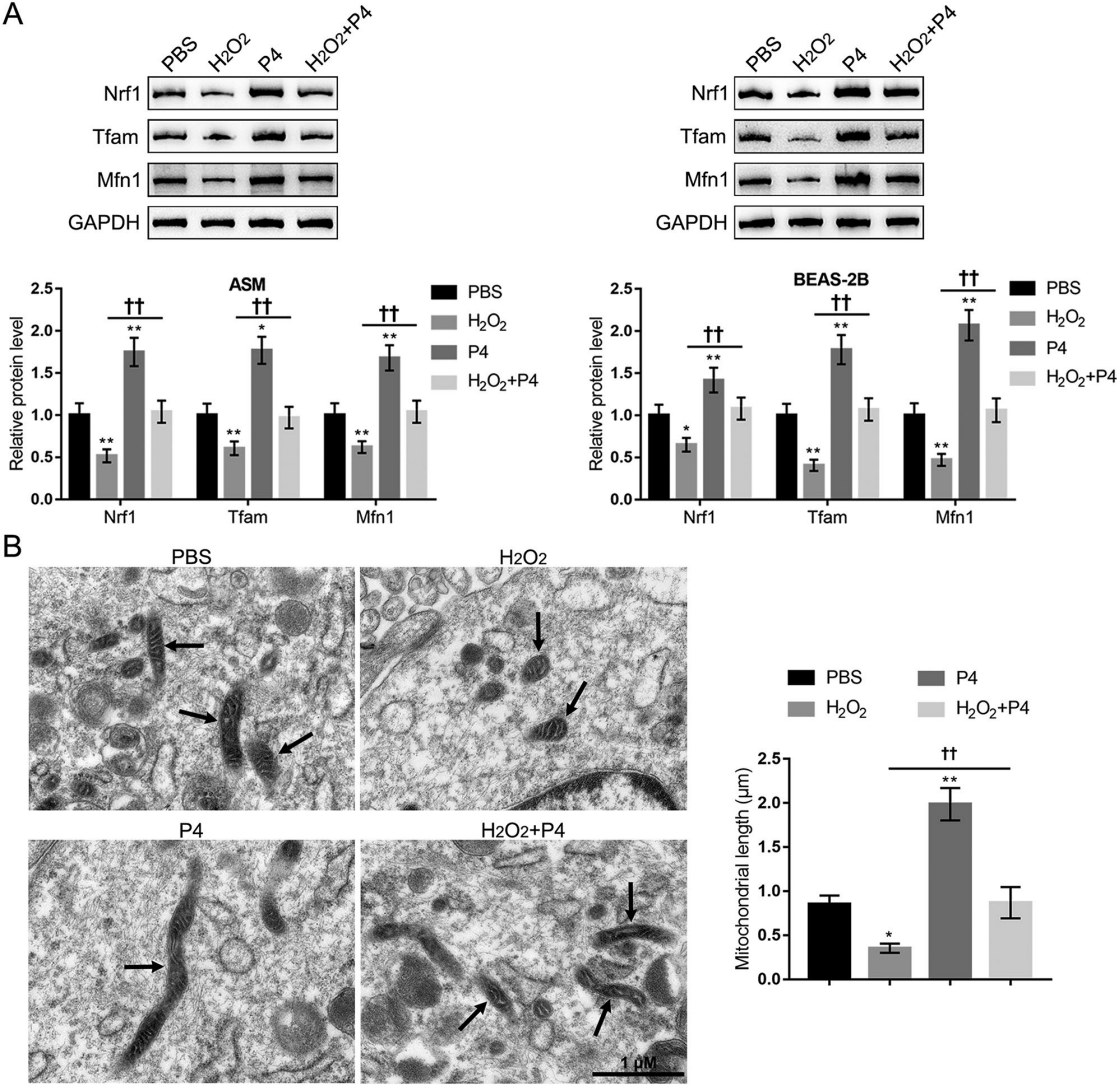
P4 modulates mitochondrial function-related proteins expression and mitochondrial length. (**A**) the protein levels of Nrf1, Tfam, and Mfn1 in ASM and BEAS-2B cells were determined using Immunoblotting. (**B**) the mitochondrial morphology changes in ASM cells were observed by TEM. *N* = 3, * *p* < 0.05, ** *p* < 0.01 compared to PBS group, †† *p* < 0.01 compared to H_2_O_2_ group

**Fig. 4:**
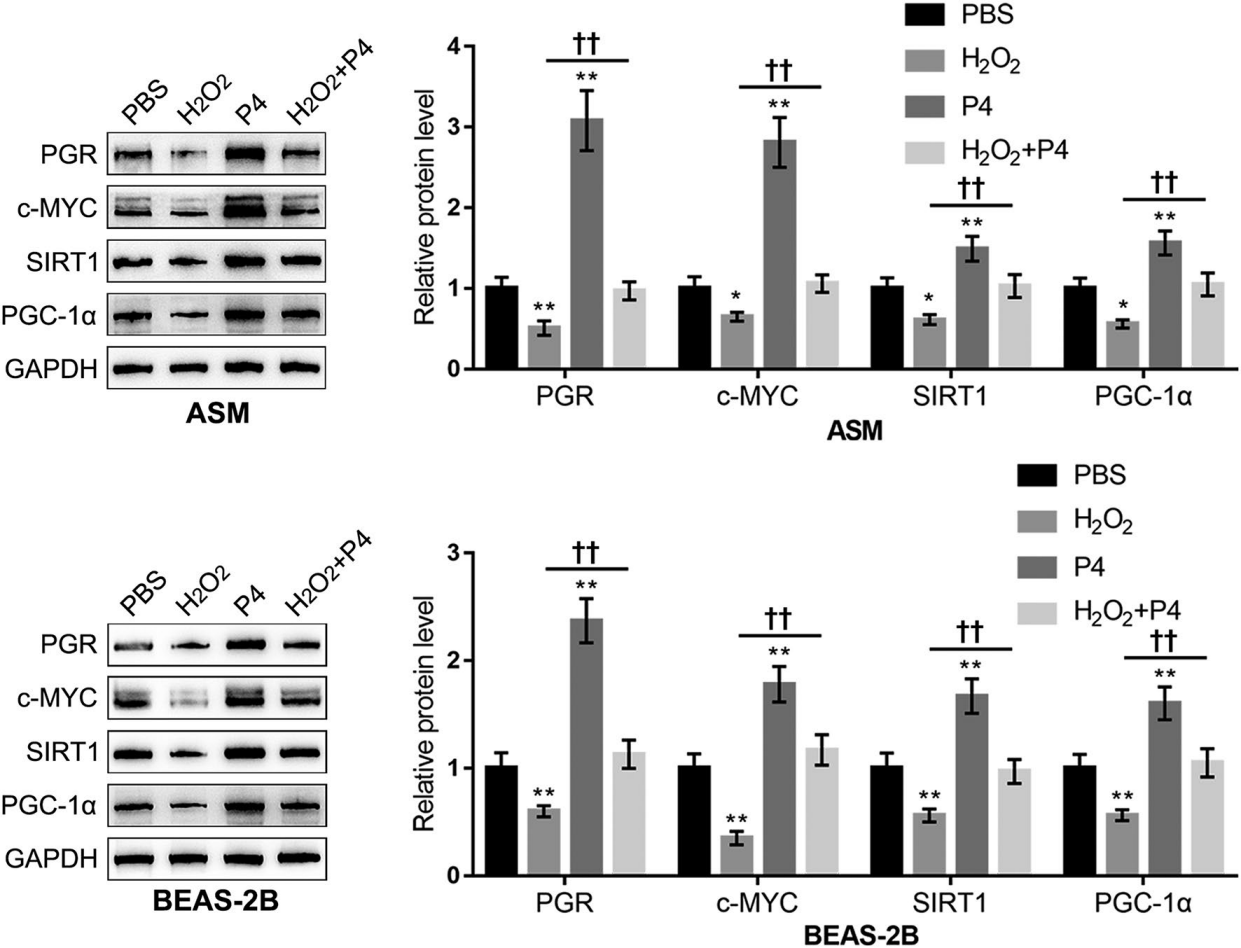
Changes in the c-MYC/SIRT1/PGC-1α cascades in P4 protection against oxidative injury. BEAS-2B and ASM cells were divided into the aforementioned four groups and the protein levels of PGR-B, c-MYC, SIRT1, and PGC-1α were determined using Immunoblotting. *N* = 3, * *p* < 0.05, ** *p* < 0.01 compared to PBS group, †† *p* < 0.01 compared to H_2_O_2_ group

Consistently, H_2_O_2_ exposure induced a reduction in the mitochondrial length, and protein levels of mitochondrial function-related proteins Nrf1, Tfam, and Mfn1, while P4 co-treatment attenuated H_2_O_2_-induced reduction in these factors (Fig. 3A and B), indicating that P4 co-treatment could attenuate H_2_O_2_-induced mitochondrial dysfunction. Furthermore, the changes in the c-MYC/SIRT1/PGC-1α cascades in epithelial cells and smooth muscle cells were also monitored. PGR-B, c-MYC, SIRT1, and PGC-1α protein contents were decreased by H_2_O_2_ exposure and increased by P4 treatment alone. Moreover, H_2_O_2_-induced reduction of those proteins’ expression was partially increased by P4 co-treatment (Fig. 4).

### c-MYC knockdown attenuated P4 protection against oxidative injury

**Fig. 5:**
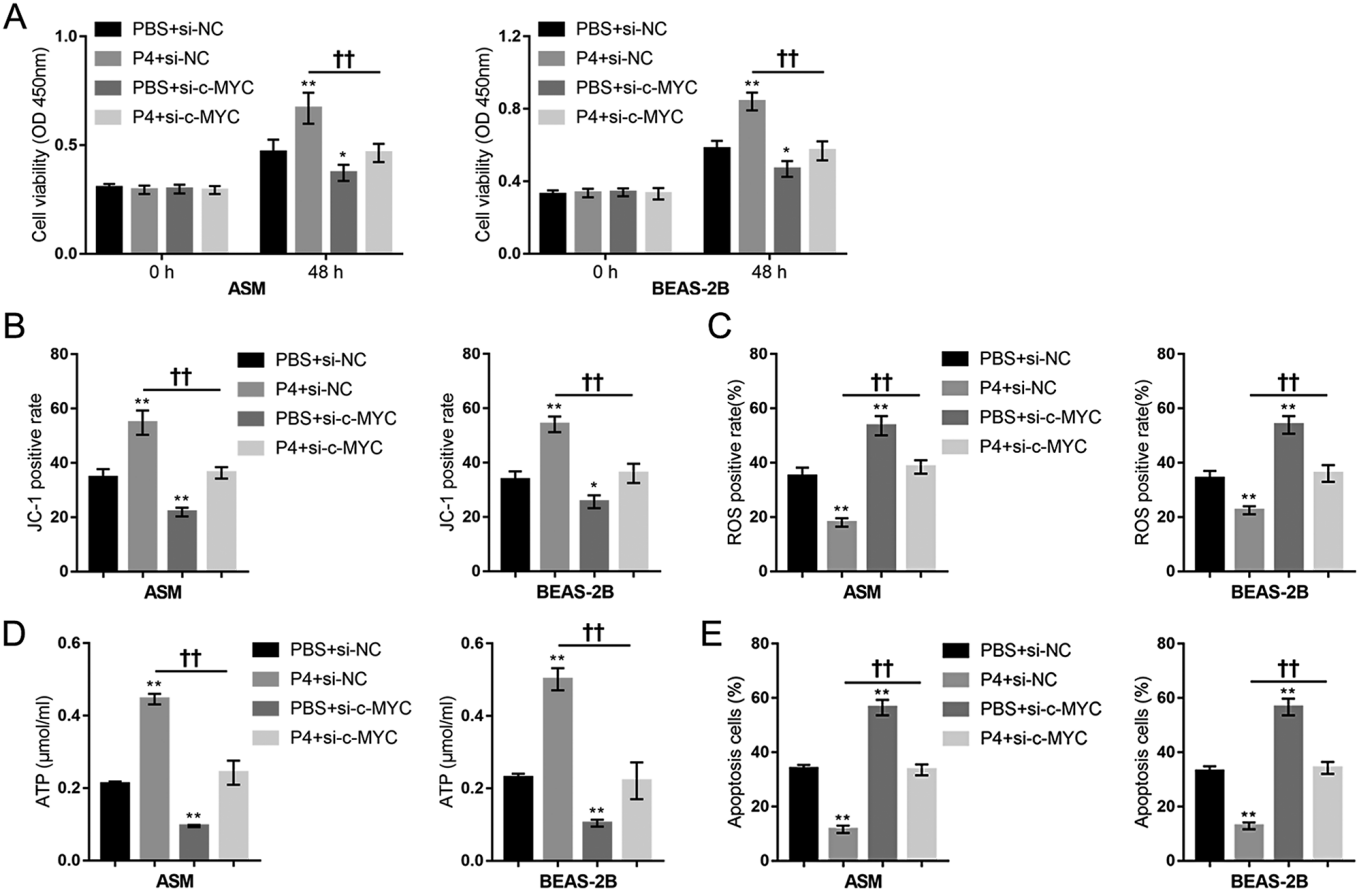
c-MYC knockdown attenuated P4 protection against oxidative injury. BEAS-2B and ASM cells were transfected with si-c-MYC, co-treated with P4, exposed to H_2_O_2_, and examined for cell viability using MTT assay (**A**); mitochondrial membrane potential by JC-1 staining (**B**); mitochondrial ROS by MitoSOX Green followed by Flow cytometry (**C**); ATP content using ATP Detection Kit (**D**); the apoptosis rate using Annexin-V/PI staining (**E**). *N* = 3, * *p* < 0.05, ** *p* < 0.01 compared to PBS + si-NC group, †† *p* < 0.01 compared to PBS + si-c-MYC group

**Fig. 6:**
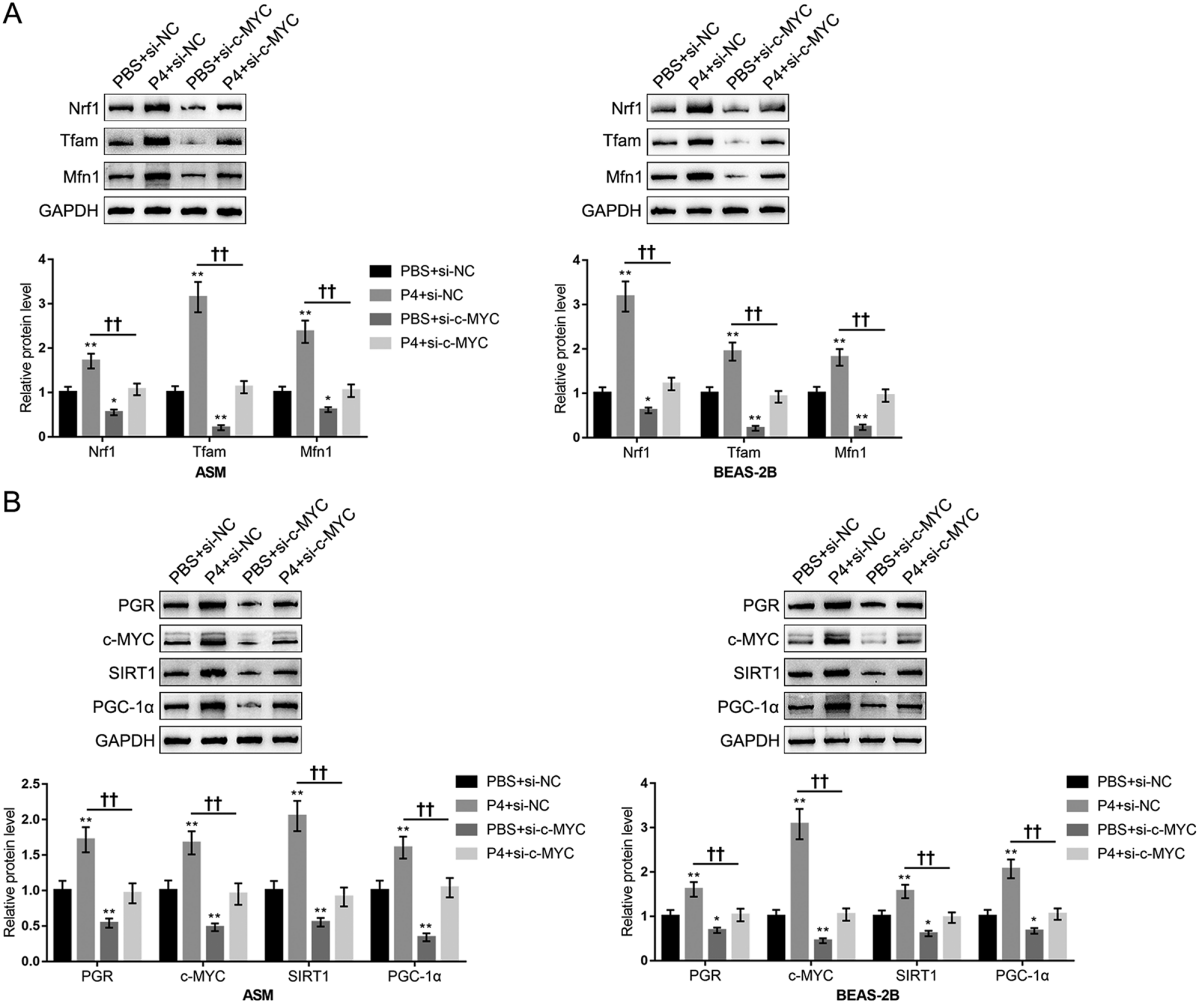
c-MYC knockdown attenuated P4 induced activation of c-MYC/SIRT1/PGC-1α pathway under oxidative injury. BEAS-2B and ASM cells were transfected with si-c-MYC, co-treated with P4, and exposed to H_2_O_2_. (**A**) the protein levels of Nrf1, Tfam, and Mfn1 using Immunoblotting; (**B**) the protein levels of PGR-B, c-MYC, SIRT1, and PGC-1α were determined using Immunoblotting. *N* = 3, * *p* < 0.05, ** *p* < 0.01 compared to PBS + si-NC group, †† *p* < 0.01 compared to PBS + si-c-MYC group

To investigate the involvement of the c-MYC/SIRT1/PGC-1α cascades, BEAS-2B and ASM cells were transfected with si-c-MYC, co-treated with P4, exposed to H_2_O_2_, and examined for oxidative injury and mitochondrial dysfunction. First, the transfection efficiency of c-MYC knockdown was verified by immunoblotting in ASM and BEAS-2B cells (Fig.S3A). Under H_2_O_2_ exposure, P4 co-treatment promoted cell proliferation (Fig. 5A), increased mitochondrial membrane potential (Fig. 5B, Fig.S4B), decreased mitochondrial ROS (Fig. 5C, Fig.S4A), increased ATP content (Fig. 5D), and decreased apoptosis (Fig. 5E, Fig.S4C). However, after knocking down c-MYC, P4 protection was partially attenuated (Fig. 5A-E). Under H_2_O_2_ exposure, P4 treatment consistently increased the protein levels of Nrf1, Tfam and Mfn1 (Fig. 6A), as well as PGR-B, c-MYC, SIRT1, and PGC-1α (Fig. 6B). Conversely, c-MYC knockdown resulted in decreased levels of these proteins. Moreover, the P4 treatment-induced elevation in these proteins was partially eliminated by c-MYC knockdown (Fig. 6A and B). To further confirm the transcriptional regulation of *c-MYC* by PGR-B, promoter-reporter assays were conducted in ASM and BEAS-2B cells. The results showed that overexpression of PGR-B significantly enhanced the transcriptional activity of the wild-type *c-MYC* promoter, whereas knockdown of PGR-B suppressed this activity in both cell types. These findings corroborate the role of PGR in directly regulating c-MYC expression (Fig. S5).

### Dynamic effects of the c-MYC/SIRT1 axis on H_2_O_2_-induced oxidative injury

**Fig. 7:**
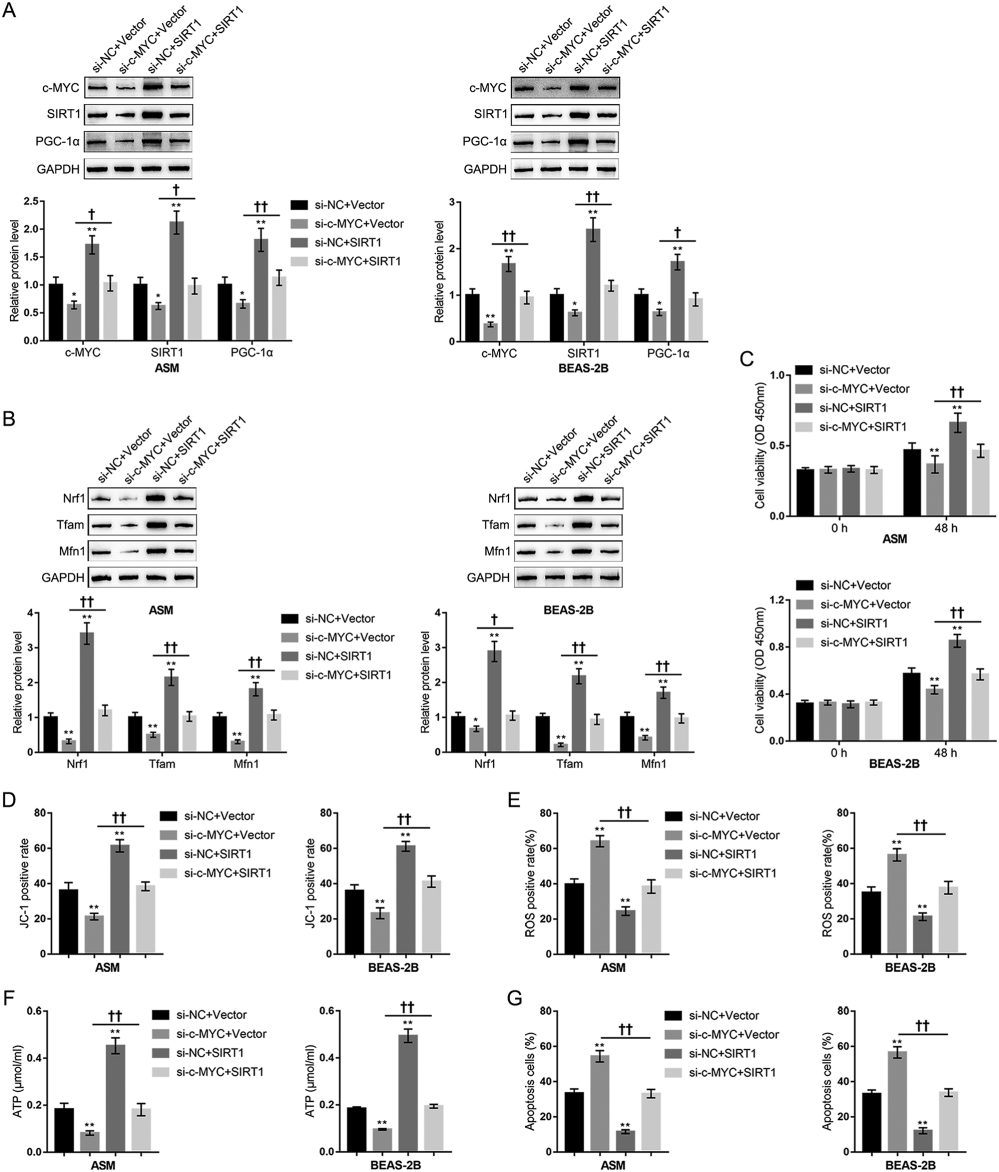
Dynamic effects of the c-MYC knockdown/SIRT1 overexpression axis on PGC-1α and its downstream proteins expression under H_2_O_2_ induced oxidative injury. BEAS-2B and ASM cells were co-transfected with si-c-MYC and SIRT1-overexpressing vector (SIRT1), exposed to H_2_O_2_, and examined for the protein levels of c-MYC, SIRT1, and PGC-1α were determined using Immunoblotting (**A**); the protein levels of Nrf1, Tfam, and Mfn1 using Immunoblotting (**B**); the cell proliferation using MTT assay (**C**); mitochondrial membrane potential by JC-1 staining (**D**); mitochondrial ROS by MitoSOX Green followed by Flow cytometry (**E**); ATP content using ATP Detection Kit (**F**); the apoptosis rate using Annexin-V/PI staining (**G**). *N* = 3, * *p* < 0.05, ** *p* < 0.01 compared to si-NC + Vector group, † *p* < 0.05, †† *p* < 0.01 compared to PBS + si-c-MYC + Vector group

Given that a c-MYC-SIRT1 feedback loop regulates cellular growth and transformation, the binding site between c-MYC and *SIRT1* promoter has been proven (Yuan et al. 2009). BEAS-2B and ASM cells were co-transfected with si-c-MYC and SIRT1-overexpressing vector (SIRT1), exposed to H_2_O_2_, and examined for the dynamic effects of the c-MYC/SIRT1 axis on H_2_O_2_-induced oxidative injury. Firstly, the overexpression efficiency of SIRT1 was confirmed by immunoblotting in ASM and BEAS-2B cells (Fig.S3B). Then, the immunoblotting results showed that c-MYC knockdown decreased the protein levels of c-MYC, SIRT1, and PGC-1α (Fig. 7A), Nrf1, Tfam, Mfn1 (Fig. 7B), whereas SIRT1 overexpression increased the protein levels of these factors (Fig. 7A-B). Deacetylation of PGC-1α leads to the transactivation of PGC-1α (Xu et al. 2021a, b). To elucidate the mechanism by which SIRT1 regulates PGC-1α, the acetylation status of PGC-1α was assessed in cells transfected with si-c-MYC or SIRT1 overexpression plasmid. Co-immunoprecipitation followed by Immunoblotting showed that SIRT1 overexpression significantly deacetylated PGC-1α (Fig. S6). The acetylation levels of PGC-1α were markedly lower in the presence of SIRT1, whereas c-MYC knockdown, likely through reduced SIRT1 expression, resulted in higher acetylation levels of PGC-1α (Fig. S6). This indicates that SIRT1 not only affects PGC-1α expression, but also directly deacetylates PGC-1α. Consequently, c-MYC knockdown aggravated H_2_O_2_-induced mitochondrial dysfunction and oxidative injury, whereas SIRT1 overexpression partially eliminated H_2_O_2_-induced changes (Fig. 7C-G). Notably, the effects of c-MYC knockdown on H_2_O_2_-induced cell signaling changes (Fig. 7A-B) and phenotypic changes (Fig. 7C-G, Fig.S7) were significantly attenuated by SIRT1 overexpression in lung epithelial cells and smooth muscle cells.

## Discussion

COPD is a progressive ailment encompassing emphysema and chronic bronchitis. COPD is characterized by the presence of damaged alveoli and inflamed airways accompanied by an excessive production of mucus. Kamil et al. (Kamil et al. 2013) highlight sex-specific and race-related disparities in COPD manifestations, yet the underlying mechanisms remain largely unexplored. P4, synthesized by the corpus luteum, is pivotal for pregnancy and menstrual cycles but has been recognized for its broader biological impacts in various tissues (Csapo et al. 1971, 1972). Low P4 levels in adult females are more responsive to O_3_ inflammatory-induced pulmonary responses (Fox et al. 1993). It is evidence that mitigates inflammation and oxidative stress across several conditions including the central nervous system, traumatic brain injury, rheumatoid arthritis, and autoimmune diseases (Sarkaki et al. 2013; Garay et al. 2017). In addition, P4 effectively reduced airway remodeling and glucocorticoid resistance in an ozone-exposed murine model (Zhang et al. 2018). Such findings imply the potential of P4 in moderating inflammation, oxidative stress, and structural alterations in COPD. In this study, the therapeutic effects of P4 were investigated in vivo and in vitro. P4 administration in a mouse model exposed to cigarette smoke (CS) mitigated the deleterious effects observed in COPD, including physiological and pathological damage, as well as inflammation, in a dose-responsive manner. Complementing these findings, our in vitro studies demonstrated that P4 can significantly counteract H_2_O_2_-induced oxidative stress and mitochondrial dysfunction in lung epithelial and smooth muscle cells.

An increasing body of evidence confirmed that sex hormones have biological and pathophysiological functions in lung diseases (Sathish et al. 2015). For example, testosterone replacement therapy may slow disease progression in COPD male patients (Baillargeon et al. 2019). Estrogen replacement induced alveolar regeneration in ovariectomy mice, suggesting estrogen may play a beneficial modulatory role in women with COPD (Massaro and Massaro 2004). As the second major endogenous female steroid hormone, P4 has been reported to inhibit chronic airway inflammation, oxidative stress, and airway remodeling in zone-exposed mice (Zhang et al. 2018). In addition, the anti-inflammatory and anti-oxidative properties of P4 have been widely reported in multiple disorders, including lung repair and recovery (Hall et al. 2016). In COPD, inflammatory cells, including eosinophils, neutrophils, macrophages, mast cells, and natural killer cells are recruited from the blood into the lungs under the direction of locally released chemotactic factors (Barnes 2017). Oxidative stress is a major driving mechanism of COPD (Barnes 2020). In this study, the reduction of total cell numbers in BALF, increased anti-oxidative enzyme activity and reduced lipid peroxidation marker MDA levels were observed in COPD mouse lung tissue after P4 therapy, suggesting that P4 reduces COPD-related inflammation and oxidative stress. Moderate- and high-concentration of P4 restored lung histology to the normal control group, evidencing that P4 treatment could reverse COPD-associated lung tissue damage (Lopez et al. 2006). Our study showed that the therapeutic potential of P4 goes beyond reducing inflammation and anti-oxidative damage. P4 protects by modulating the c-MYC/SIRT1/PGC-1α pathway. P4 therapy restores the expression of c-MYC, SIRT1, PGC-1α, PGR-B, Nrf1, Tfam, and Mfn1 after CS exposure. These proteins regulate mitochondrial biogenesis, function, and redox state (Poulose and Raju 2014). Our results support earlier studies showing CS exposure impairs mitochondrial function and increases oxidative stress in COPD (Lyons et al. 1958; Miro et al. 1999). Therefore, the cellular and molecular mechanisms of P4 therapy might be related to alleviating oxidative stress.

Oxidative stress is a major driving mechanism in the pathogenesis of COPD (Barnes 2020). P4 has been shown to protect several types of cells against oxidative injury (Robertson et al. 2006; Gaignard et al. 2016; Cai et al. 2015; Qin et al. 2015; Gonzalez Deniselle et al. 2012; Feng et al. 2014; Dai et al. 2013). Under excessive oxidative stress, epithelial cells induce the production of inflammatory mediators and ROS, causing damage to cellular components, including DNA, proteins, and lipids, and subsequently resulting in apoptotic pathway activation impacting the layer of epithelial cells. Mitochondria are the main target of ROS-caused injury to epithelial cells and the major producer of ROS in cells generated by oxidative phosphorylation (Sachdeva et al. 2019; Liu and Chen 2017). In this study, ROS production was significantly elevated in BEAS-2B and ASM cells exposed to H_2_O_2_, along with the loss of mitochondrial membrane potential and cell viability, indicating H_2_O_2_-induced mitochondrial dysfunction and oxidative injury. Consequently, dysfunction of mitochondria results in the reduction of cellular ATP levels. Contrariwise, P4 co-treatment partially attenuated H_2_O_2_-induced changes, suggesting that P4 indeed plays a protective role in airway epithelium against oxidative injury. Interestingly, P4 inhibited the cellular antioxidant effect and promoted H_2_O_2_-induced ROS and NO production in arterial endothelial cells (Yuan et al. 2016). These contradictory effects of P4 in response to H_2_O_2_ may be associated with different cell types and cell status.

Regarding the involved pathways, in response to H_2_O_2_ stimulation, c-MYC and SIRT1 levels were significantly downregulated, whereas P4 pre-treatment partially elevated the levels of c-MYC and SIRT1, consistent with previous studies (Daniel et al. 2010; Moore et al. 2012). c-MYC induces genes related to mitochondrial structure and function, thereby exerting a critical effect on the regulation of mitochondrial biogenesis (Poulose and Raju 2014). Thus, c-MYC might participate in P4 protection against oxidative injury and mitochondrial dysfunction. As speculated, c-MYC knockdown markedly abolished P4 protection on BEAS-2B and ASM cells against H_2_O_2_-induced mitochondrial damages. Notably, the levels of SIRT1 also altered in response to P4 treatment and/or c-MYC knockdown. A c-MYC-SIRT1 feedback loop has been previously reported that c-MYC induces the transcription of SIRT1 by targeting its promoter while SIRT1 induces the deacetylation of c-MYC to promote its stability and transactivation (Menssen et al. 2012; Mao et al. 2011), suggesting a mechanism of SIRT1 mediated c-MYC regulation. Herein, it was also found that c-MYC promotes SIRT1 transcription to mediate P4 protection against oxidative damage.

P4 affects a cell through its nuclear receptor PGR, which has two main isoforms: PGR-A and PGR-B (Kowalik et al. 2013). PGR-A often functions as a transcriptional repressor of PGR-B and other steroid receptors (Conneely and Lydon 2000; Li and O’Malley 2003; Brosens et al. 2004; Giangrande and McDonnell 1999), including estrogen and glucocorticoid receptors. PGR-B acts as a stronger transcriptional activator than PGR-A, More genes are uniquely regulated by ligandized PGR-B than by PGR-A, and relatively few genes are regulated by both isoforms (Jacobsen et al. 2005; Richer et al. 2002). The promoter c-MYC gene contained the progesterone response elements (Moore et al. 1997a, b). Activated PGR-B is essential for P4-induced upregulation of c-MYC expression (Shen et al. 2001). Our findings confirm that PGR-B facilitates c-MYC transcription in ASM and BEAS-2B cells. This transcriptional activation plays a significant role in the protective effects of P4 in oxidative stress and mitochondrial dysfunction. In addition to PGR, several steroid hormone receptors have been reported to affect COPD or other lung diseases (Ambhore et al. 2021). Estrogen regulates various lung functions through interactions with estrogen receptors (ERs), including ERα, ERβ and GPR30, potentially influencing lung development, inflammation, and disease outcomes (Fuentes and Silveyra 2018). The GPR30 mediates rapid non-genomic estrogen signals, including EGFR, MAPK and PI3K pathway (Prossnitz and Arterburn 2015). Targeting EGFR/MAPK signaling may represent a novel therapeutic panacea for treating COPD (Xu et al. 2023; Vallath et al. 2014). Future studies should further elucidate the contributions of these receptors to fully understand their roles in COPD pathogenesis and therapy.

**Fig. 8:**
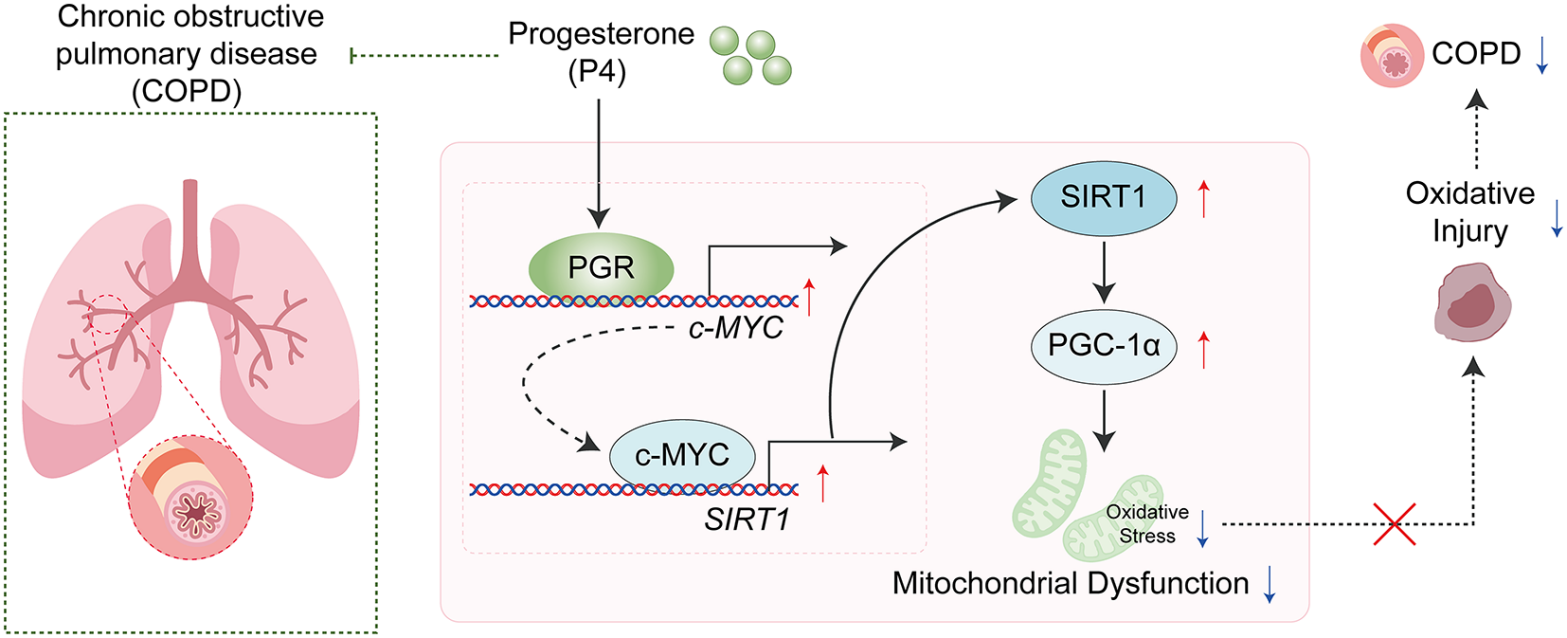
The schematic diagram of the protective role of progesterone in COPD

Reportedly, in response to pathological stress, although PGC-1α activity is decreased, c-MYC is responsible for the regulation of cardiac metabolism and mitochondrial biogenesis (Ahuja et al. 2010). As aforementioned, SIRT1 also mediates the deacetylation of PGC-1α to activate PGC-1α, subsequently regulating the expression of downstream mitochondrial function-related proteins such as Nrf1, Nrf2, Tfam, Mfn1, Drp1, etc. (Dabrowska et al. 2015). PGC-1α requires both AMPK phosphorylation and SIRT1 deacetylation for its complete activation. To further elucidate the role of SIRT1 in regulating PGC-1α in BEAS-2B and ASM cells, the acetylation status of PGC-1α in the context of c-MYC and SIRT1 expression was investigated. Reduced acetylation levels of PGC-1α were observed in cells overexpressing SIRT1, while c-MYC knockdown reduced SIRT1 levels, thereby increasing PGC-1α acetylation. These results align with previous studies indicating that SIRT1-mediated deacetylation is crucial for PGC-1α activation (Xu et al. 2021a, b; Ning et al. 2018). Here, under H_2_O_2_ stimulation, in c-MYC knockdown lung epithelial cells and smooth muscle cells, SIRT1, PGC-1α, and downstream Nrf1, Tfam, and Mfn1 levels were significantly decreased; whereas after overexpressing SIRT1, SIRT1, PGC-1α, Nrf1, Tfam, and Mfn1 levels were partially increased. Consistent with the molecular changes, H_2_O_2_-induced oxidative damages and mitochondrial dysfunctions were aggravated by c-MYC knockdown and attenuated by SIRT1 overexpression, indicating that P4 up-regulates c-MYC/SIRT1 axis and exerts the protective effects through the mitochondrial signaling pathway. Correspondingly, in the CS-induced mouse model, inhalation of P4 also up-regulated the c-MYC/SIRT1/ PGC-1α axis in lung tissues and alleviated lung inflammation and injury. Regarding possible mechanisms, P4 exerts its therapeutic effects in COPD through multiple facets. Studies have shown that c-MYC is involved in mitochondrial biogenesis and metabolism (Morrish and Hockenbery 2014), SIRT1 regulates mitochondrial function and antioxidant responses (Li 2013), and PGC-1α plays a central role in mitochondrial regeneration and oxidative stress resistance (St-Pierre et al. 2003). Therefore, P4 could boost the expression of c-MYC, a transcription factor known to play a pivotal role in cellular metabolism and growth. The upregulation of c-MYC, in turn, could stimulate the expression of SIRT1, a deacetylase implicated in stress responses and mitochondrial function. The activation of SIRT1 could subsequently promote the deacetylation and activation of PGC-1α, a master regulator of mitochondrial biogenesis and antioxidant defense. This sequence of events suggests a comprehensive mechanism by which P4 could ameliorate the symptoms and progression of COPD by improving mitochondrial function and reducing oxidative injury in lung tissues (Fig. 8).

In conclusion, this study uniquely demonstrates the novel therapeutic potential of P4 in ameliorating cigarette smoke-induced COPD by enhancing mitochondrial function and energy metabolism in epithelial cells and airway smooth muscle cells. Unlike previous studies that primarily focused on P4’s role in reproductive health, our study elucidates the mechanisms by which P4 activates the c-MYC/SIRT1/PGC-1α axis, thereby promoting mitochondrial function and reducing oxidative stress, representing a significant advancement in understanding the metabolic and molecular pathways involved in COPD and offering a new avenue for potential treatment strategies.

### Electronic supplementary material

Below is the link to the electronic supplementary material.

Supplementary Material 1

## Data Availability

All data and materials are available.

## Abbreviations

ASM cells: Airway smooth muscle cells
COPD: Chronic obstructive pulmonary disease
CS: Cigarette smoke
P4: Progesterone
ROS: Reactive oxygen species
MDA: Malondialdehyde
CAT: catalase
SOD: Superoxide dismutase
Drp1: Dynamin-related protein 1
Fis1: Mitochondrial fission 1 protein
Mfn1 and Mfn2: Mitofusin
OPA1: Optic nerve atrophy 1
Tfam: Mitochondrial transcription factor A
PGR-B: Progesterone receptor-B
SIRT1: Sirtuin 1
PGC-1α: peroxisome proliferators activated receptor gamma co-activator 1 alpha
ΔΨm: Mitochondrial membrane potential
TEM: Transmission electron microscope
