# Supplementary material for: Progesterone (P4) ameliorates cigarette smoke-induced chronic obstructive pulmonary disease (COPD)
Source: Mol Med. 2024 Aug 13;30:123. doi: 10.1186/s10020-024-00883-y (PMC11323532; doi:10.1186/s10020-024-00883-y)
Supplement: Supplementary file 1 — Supplementary Material 1 [file 10020_2024_883_MOESM1_ESM.docx]

**Fig. S1 Evaluation of the physical safety of P4 administration** A total of 24 male mice were divided into four groups: control, low-dose P4, moderate-dose P4, and high-dose P4 and subjected to nebulized administration of corresponding doses of P4 for 8 weeks. (A) The body weight and the histopathological changes in mouse lung (B), liver (C), and spleen (D) were evaluated using H&E staining. N=6.


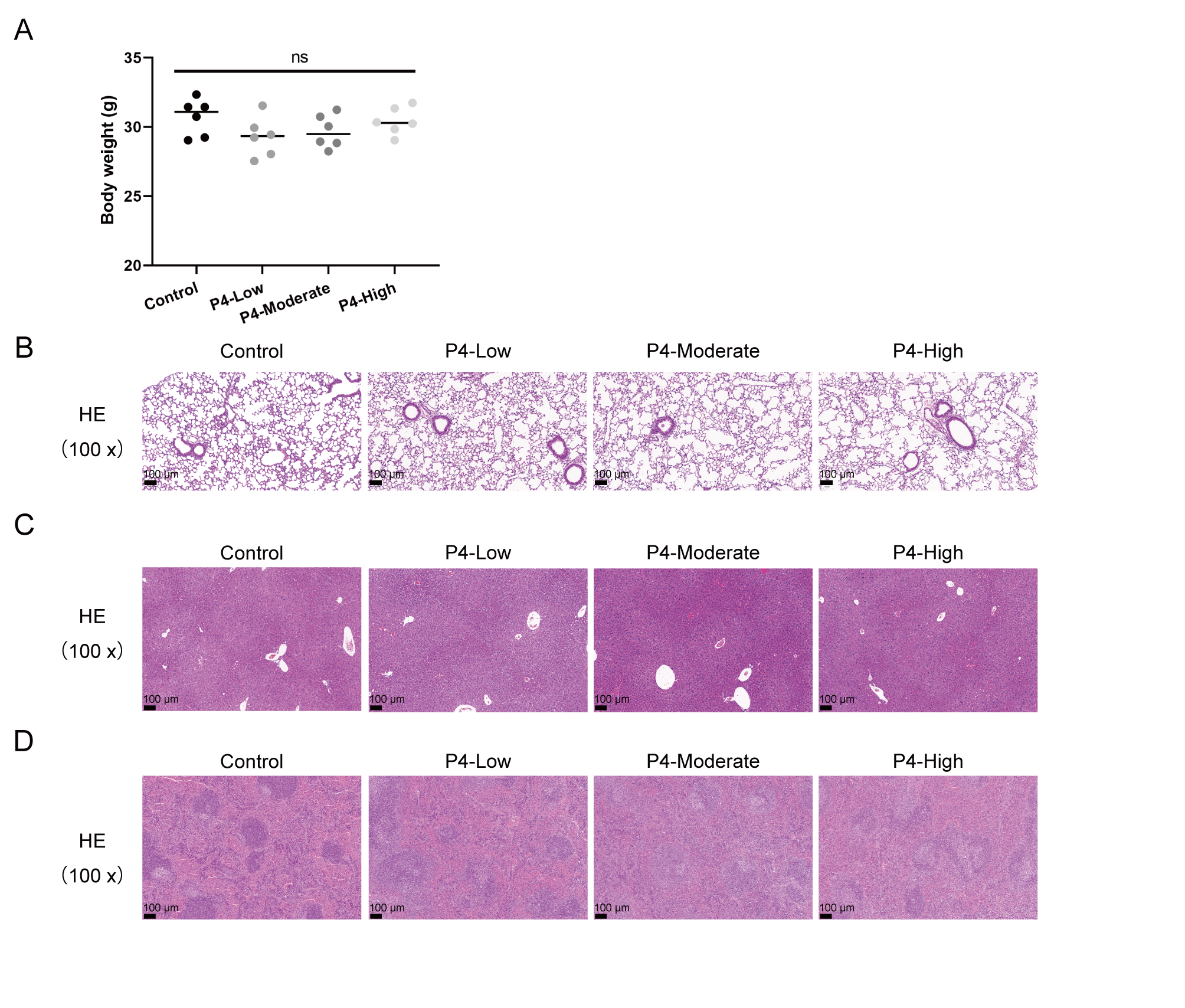

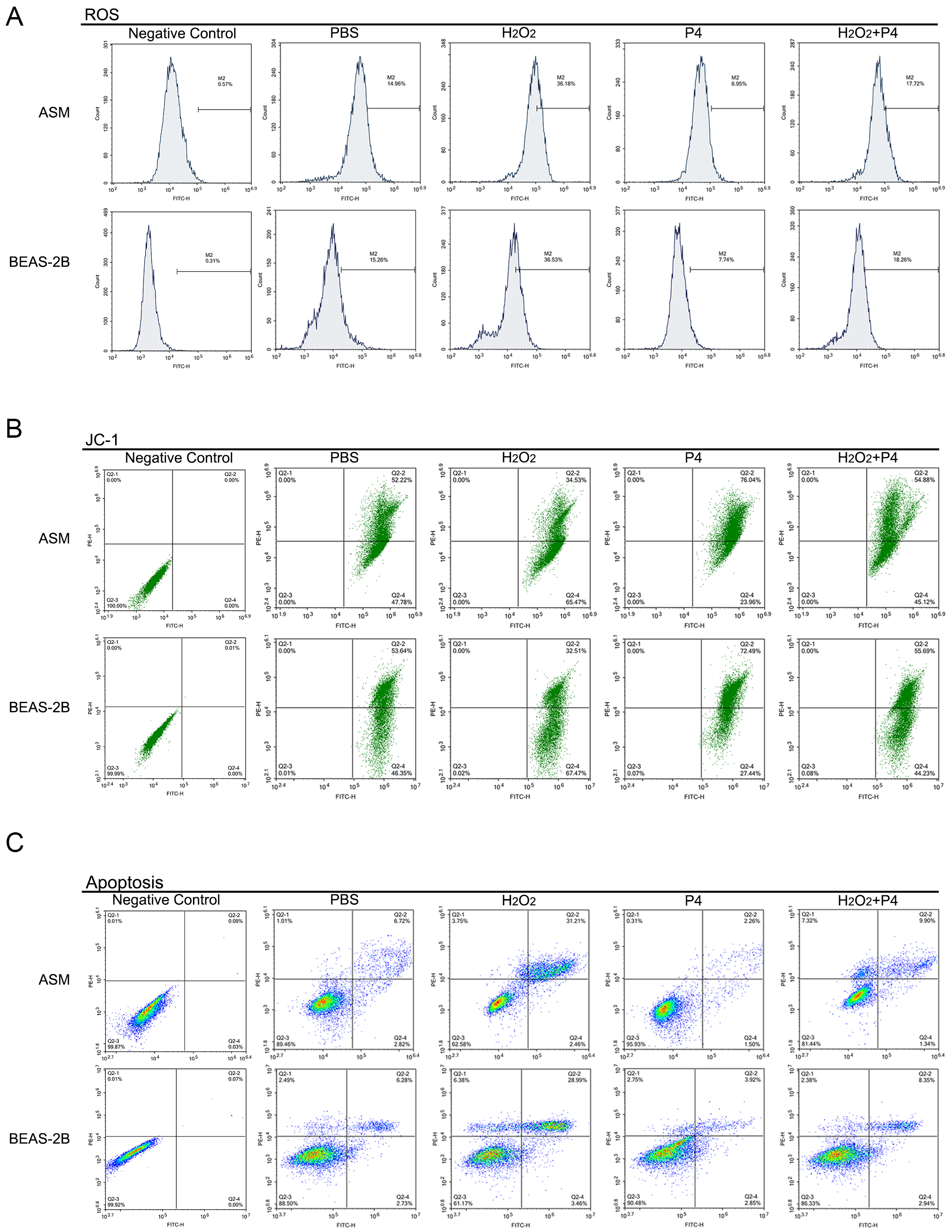


**Fig.S2 The flow cytometry images of fig.2.** BEAS-2B and ASM cells were divided into four groups: PBS (control), H_2_O_2_, P4, and H_2_O_2_ + P4; cells were treated or co-treated as described and examined for (A) mitochondrial ROS were detected by MitoSOX Green followed by Flow cytometry. (B) mitochondrial membrane potential by JC-1 staining. (C) apoptosis by Annexin-V/PI staining.

**
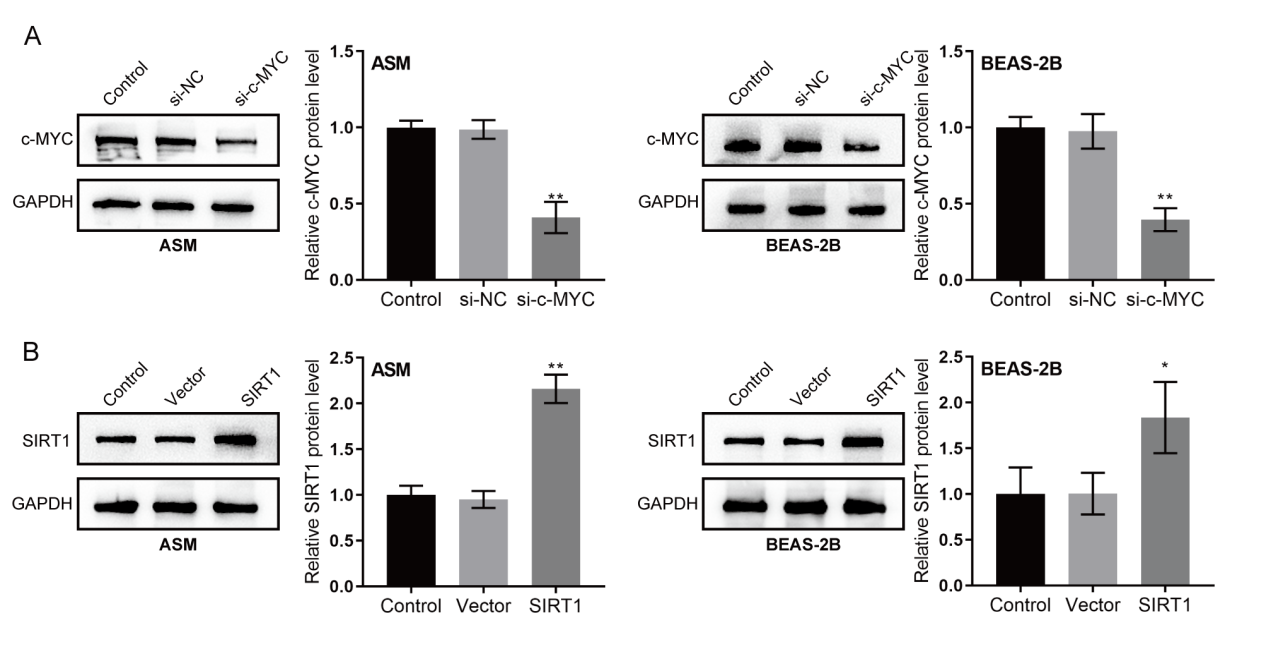
**

**Fig. S3 The transfection efficiency in ASM and BEAS-2B cells was validated using Immunoblotting**. (A) The transfection efficiency of si-c-MYC. (B) The transfection efficiency of SIRT1 overexpression. N=3. * p <0.05, ** p <0.01. compared to the si-NC or vector group.


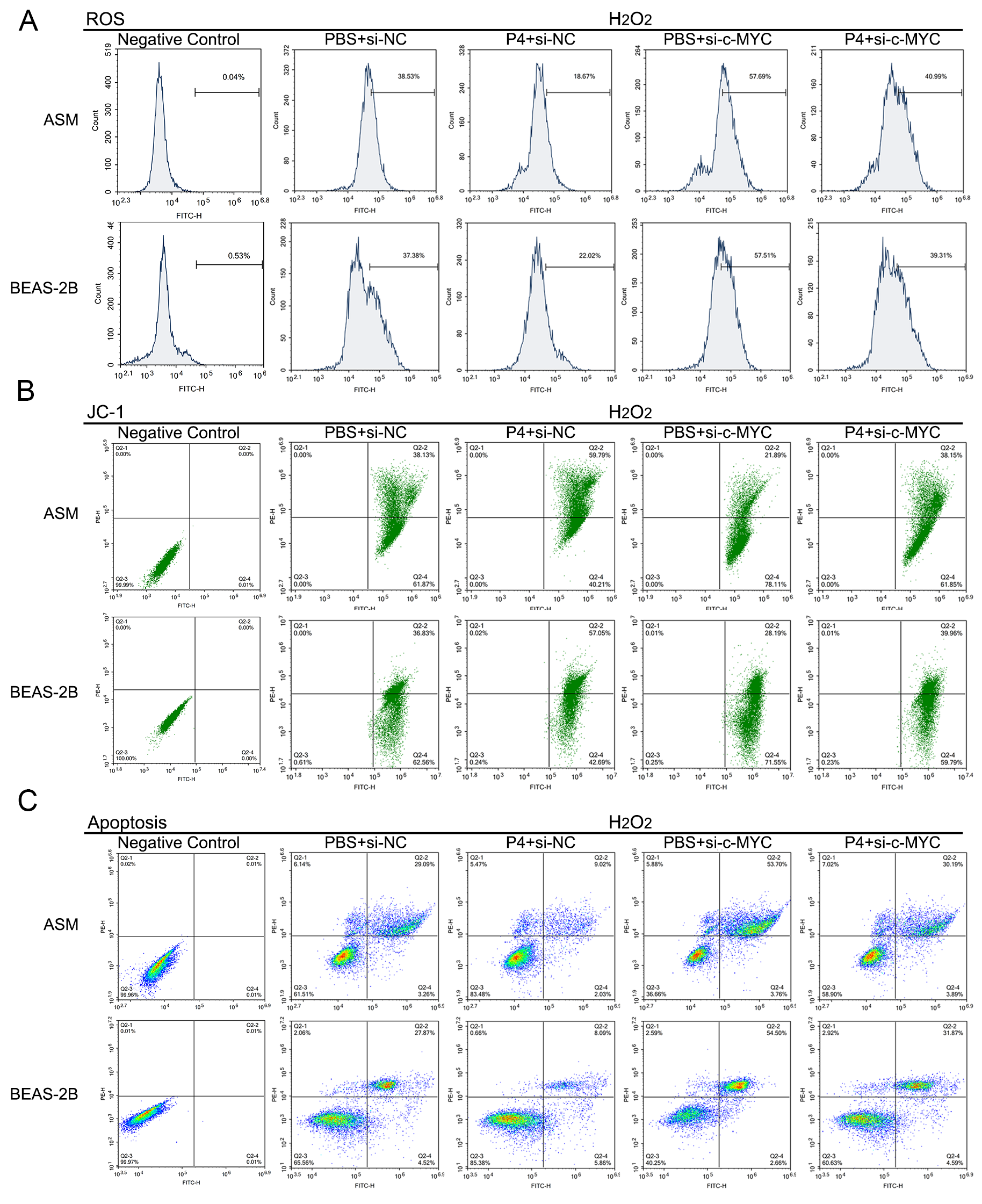


**Fig.S4 The flow cytometry images of fig.5.** BEAS-2B and ASM cells were transfected with si-c-MYC, pre-treated with P4, exposed to H_2_O_2_, and examined for (A) mitochondrial ROS were detected by MitoSOX Green followed by Flow cytometry. (B) mitochondrial membrane potential by JC-1 staining. (C) apoptosis by Annexin-V/PI staining.


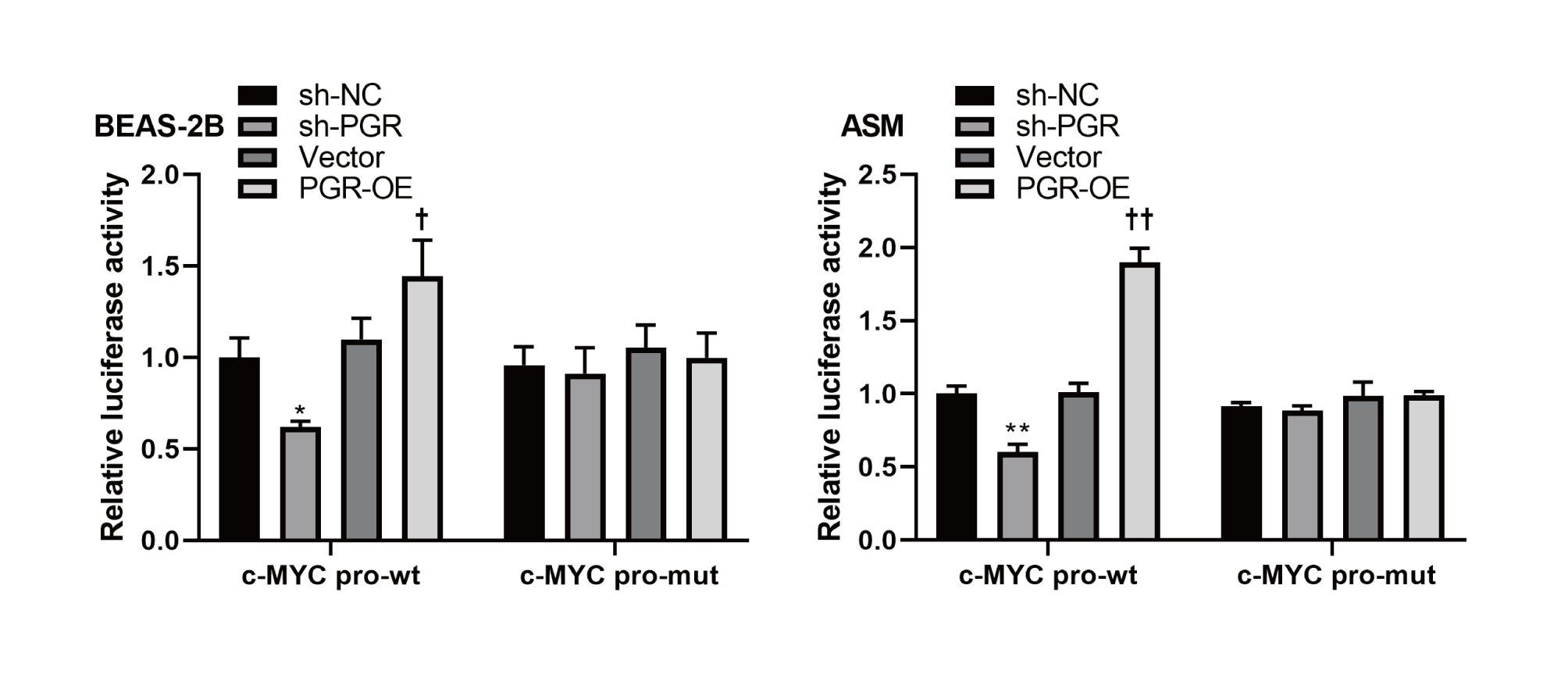


**Fig.S5 The transcription activity of PGR-B on *c-MYC* promoter was determined by Luciferase reporter assay.**  A PGR-B-overexpressing plasmid (PGR-OE) or plasmid containing short hairpin RNA targeting PGR-B (sh-PGR) were constructed. The psiCheck-2 reporter vectors, which contained either the wild-type (wt) or mutant (mut) c-Myc promoter fragment (c-MYC pro-wt and c-MYC pro-mut) were also constructed. Then, the reporter vectors were co-transfected with PGR-OE or sh-PGR into ASM and BEAS-2B cells. The relative luciferase activity was quantified using a Dual-Luciferase Reporter Assay System. * p<0.05, ** p<0.01 compared to sh-NC group; † p <0.05, †† p <0.01 compared to vector group.


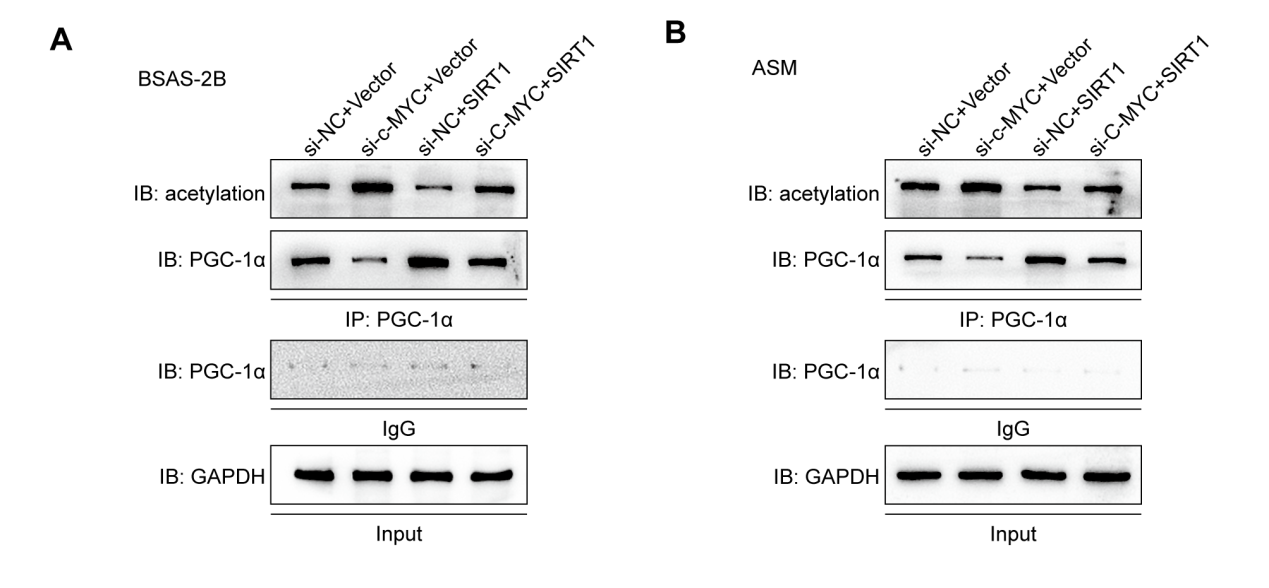


**Fig.S6 The acetylation status of PGC-1α in cells transfected with si-c-MYC and SIRT1 was determined using co-immunoprecipitation (Co-IP) followed by Immunoblotting.** Immunoprecipitation of PGC-1α from lysates of ASM and BEAS-2B cells transfected with si-c-MYC or SIRT1 overexpression vector followed by immunoblotting with anti-PGC-1α or anti-acetyl-lysine antibodies.

**
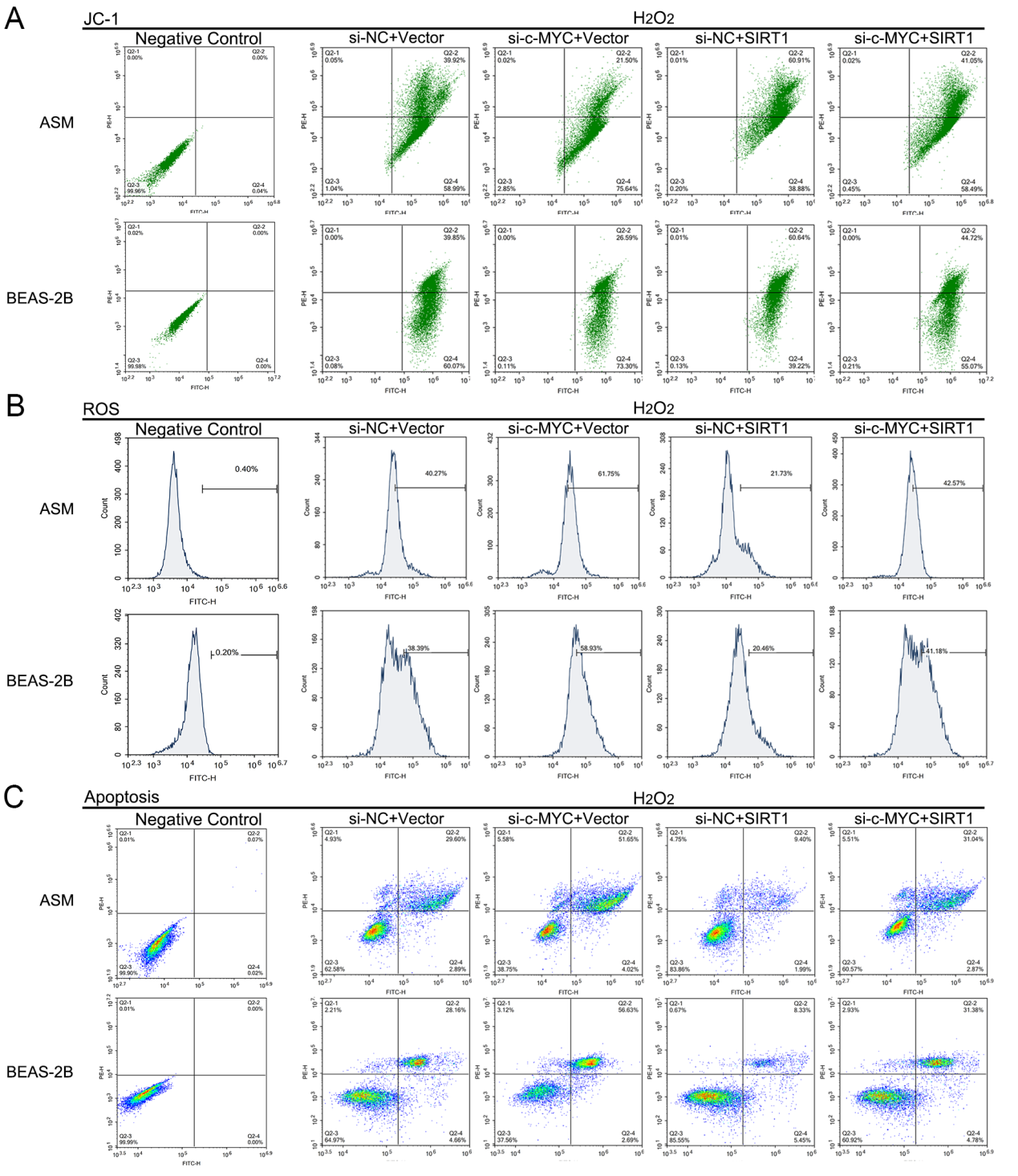
**

**Fig.S7 The flow cytometry images of fig.7.** BEAS-2B and ASM cells were co-transfected with si-c-MYC and SIRT1-overexpressing vector (SIRT1), exposed to H_2_O_2_, and examined for (A) mitochondrial membrane potential by JC-1 staining. (B) mitochondrial ROS were detected by MitoSOX Green followed by Flow cytometry. (C) apoptosis by Annexin-V/PI staining.

Table S1 Sequences of siRNA and vector construction

| Name | Forward/sense 5’-3’ | Reverse/antisense 5’-3’ |
| --- | --- | --- |
| Si-NC | UUCUCCGAACGUGUCACGUTT | ACGUGACACGUUCGGAGAATT |
| Si-c-MYC | GUAUUAUAGGUACUAUAAATT | UUUAUAGUACCUAUAAUACTT |
| Si-SIRT1 | GGUUCAUUUGUAUGAUAAATT | UUUAUCAUACAAAUGAACCTT |
| c-MYC overexpression  pcDNA3.1 | ctagcgtttaaacttaagcttATGGATTTTTTTCGGGTAGTGG | tgctggatatctgcagaattcTTACGCACAAGAGTTCCGTAGC |
| SIRT1  overexpression  pcDNA3.1 | ctagcgtttaaacttaagcttATGGCGGACGAGGCGGCC | tgctggatatctgcagaattcCTATGATTTGTTTGATGGATAGTTCATG |
| c-myc pro wt | aattctaggcgatcgctcgagTGCGGCAAAGGCCTGGAG | attttattgcggccagcggccgcAGATAAAGCCCCGAAAACCG |
| c-myc pro mut | GGGAATATtgtaatataATTAAATATAGATCATTTCAGGGAGCAA | tatattacaATATTCCCTCGGGATTTTTTATTTT |
| PGR-B overexpression | ctagcgtttaaacttaagcttATGACTGAGCTGAAGGCAAAGG | tgctggatatctgcagaattcTCACTTTTTATGAAAGAGAAGGGGT |
| Sh-PGR-B | GATCCGGAGTTTGTCAAGCTTCAAGTCTCGAGACTTGAAGCTTGACAAACTCCTTTTTG | AATTCAAAAAGGAGTTTGTCAAGCTTCAAGTCTCGAGACTTGAAGCTTGACAAACTCCG |
| Sh-NC | GATCCGCAGATGAAGGCACGGTCACGCTCGAGCGTGACCGTGCCTTCATCTGCTTTTTG | AATTCAAAAAGCAGATGAAGGCACGGTCACGCTCGAGCGTGACCGTGCCTTCATCTGCG |
